# Effects of quercetin, baicalein, azithromycin, and their combination on biofilm formation, virulence factors and gene expression associated with *Pseudomonas aeruginosa* quorum sensing

**DOI:** 10.1007/s11033-026-11876-x

**Published:** 2026-05-22

**Authors:** Valentina Parra Rodríguez, Vanessa Gómez, Ludy Cristina Pabón, Patricia Hernández-Rodríguez

**Affiliations:** Escuela de Ciencias Básicas y Aplicadas, Universidad de la Salle, Bogotá, Colombia

**Keywords:** Pseudomonas aeruginosa, Biofilms, Quorum sensing, Flavonoids, Combination drug therapy

## Abstract

**Background:**

*Pseudomonas aeruginosa* is a Gram-negative opportunistic pathogen commonly associated with acute and chronic hospital-acquired infections. Its ability to form biofilms, regulated in part by quorum sensing, contributes to its persistence and resistance. Classified as a critical priority pathogen by the World Health Organization, there is an urgent need for new therapeutic strategies. In this study, we evaluated the effects of quercetin, baicalein and azithromycin, alone and in combination, on biofilm formation, virulence factor production, and quorum sensing gene expression in *P. aeruginosa* PAO1.

**Methods and Results:**

The minimum inhibitory concentration of each compound was measured. The effect of each compound and their combinations on biofilm formation, elastases, pyocyanin and rhamnolipids were evaluated by spectrophotometric assays, and on *lasR* and *mvfR* gene expression by RT-qPCR. The minimum inhibitory concentrations of quercetin, baicalein and azithromycin were > 250, 62, and 16 µg/mL, respectively. The individual compound with the lowest percentage of biofilm formation was quercetin, followed by azithromycin and baicalein with 33%, 48%, and 51%, and the best combination was azithromycin-baicalein with 35%. Azithromycin and the mentioned combination showed the lowest production of elastases, pyocyanin and rhamnolipids (39% and 34%; 8% and 13%; 19% and 16%, respectively) and resulted in *lasR* and *mvfR* gene expression levels of 32% and 34%.

**Conclusions:**

The combination of azithromycin-baicalein showed inhibitory effects on biofilm formation, virulence factors and gene expression of *lasR* and *mvfR*. These findings highlight the potential of combining natural products with antibiotics as a promising strategy to attenuate virulence and disrupt quorum sensing-regulated behaviors in *P. aeruginosa*.

## Introduction

*Pseudomonas aeruginosa* (*P. aeruginosa*) is a common agent of hospital-acquired infections; it is estimated to have a prevalence of 7.1% to 7.3% among all healthcare-associated infections [1]. An infection by this pathogen leads to diseases with a high mortality rate in patients with ventilator-associated pneumonia (VAP), reaching values between 32% and 43%, and from 43.2% to 58.8% in patients with bloodstream infections [2], as well as in patients with cystic fibrosis (CF), cancer, severe burns, and other forms of immunocompromise. This microorganism can survive in water, on different surfaces, and on medical devices because of its binding factors (pili and biofilms), its great metabolic versatility and its high adaptability to environmental changes. *P. aeruginosa* shows three types of antibiotic resistance that make it difficult to control with currently available drugs. This can be explained by its ability to form biofilms on abiotic and medical surfaces and by its survival strategies, related to virulence factors, which allow it to avoid the host’s immune response [3]. Biofilm formation is regulated by Quorum Sensing (QS), which takes part in several common bacterial behaviors, including virulence factor expression, mainly involved in the success of infection. The QS of *P. aeruginosa* is regulated by three already characterized systems, LasL/LasR, RhlL/RhlR and Pqs/PqsR, which manage independent but interrelated pathways [4].

The LasL/LasR system induces the expression of genes linked to various virulence factors, including hemolysins, proteases, elastases A andB, and exotoxin-A, which play a crucial role in bacterial pathogenesis and biofilm formation. Factors such as elastase A and B (LasA and LasB) are produced to destroy elastin in lung tissue and blood vessels, affecting lung function by causing hemorrhage [5].

The RhlL/RhlR system controls the expression of cytotoxic virulence factors such as LecA - LecB, as well as swarming, twitching and type IV pili bacterial motilities. This type of motility, regulated by the Rhl process in a minimal iron-limited medium, is necessary to assemble bacteria in monolayers that form micro-colonies. Likewise, this process controls rhamnolipid production, which play multiple roles in the formation and structure of *P. aeruginosa* biofilms, such as the formation of micro-colonies, the development of mushroom-shaped structures, and the maintenance of open channel structures necessary for the circulation of nutrients [6].

The Pqs/PqsR system, also known as the multiple virulence-factor regulator MvfR, is necessary in the complete virulence of *P. aeruginosa* because it regulates the activity of the factors through the synthesis of 4-hydroxy-2-alkylquinolines (HAQs). This process controls gene transcription not regulated by RhlL/RhlR, contributing to virulence, which, in addition, is enhanced by positively regulating LasL/LasR and RhlL/RhlR-dependent genes [7]. This interaction is reflected in pyocyanin production, regulated by RhlL/RhlR and MvfR, a virulence factor considered critical for *P. aeruginosa* lung infections in mice and airway colonization in cystic fibrosis patients. This factor helps to promote eDNA release and, together with it, facilitates cell-to-cell communication in biofilms [8].

The interactions of the processes that regulate the QS of *P. aeruginosa* result in resistance to conventional drugs, making it necessary to develop new therapeutic strategies for the control of infections caused by this bacterium. An alternative approach is to combine antibiotics with natural compounds that can inhibit the QS and enhance the activity of antibiotics by changing or blocking the resistance mechanism and making bacteria susceptible to the antibiotic by reducing the effective dose [9].

Flavonoids are a diverse group of plant secondary metabolites that have been demonstrated to act as potent inhibitors of QS by interfering with bacterial communication and virulence factors without necessarily affecting cellular growth [10, 11]. Quercetin and baicalein are notable examples of bioactive molecules, as they act as competitive antagonists of QS receptors such as LasR and MvfR [11, 12]. In the case of quercetin, studies indicate that it represses the expression of key QS-regulated genes (*lasI/R* and *rhlI/R*) and reduces the synthesis of pyocyanin and elastase, possibly through regulation of the *vfr* gene [11, 13]. Baicalein, in contrast, strongly inhibits QS regulatory circuits (las, rhl and pqs) and significantly reduces the production of signaling molecules (3-oxo-C12-HSL and C4-HSL). These effects have been validated through computational molecular docking studies and in vivo models, including *Caenorhabditis elegans* and murine systems [11]. In combination therapy, flavonoids have been employed to enhance the susceptibility of bacterial biofilms to conventional antibiotics. Successful combinations have been reported for quercetin with tobramycin and amikacin [11, 14], as well as for baicalein with levofloxacin, amikacin, ceftazidime, imipenem, and colistin [15].

However, no specific studies have been reported evaluating the combination of quercetin or baicalein with azithromycin. Azithromycin has been the subject of synergy studies with other natural compounds, particularly in combination with curcumin [11, 16], vitexin [17], and andrographolide [18]. Additionally, pharmacological combinations of azithromycin with ciprofloxacin have been shown to enhance bacterial eradication in mature biofilms [19]. For the above reasons, this study evaluated the effects of quercetin, baicalein, azithromycin, and their combinations on biofilm formation, virulence factors and on the expression of QS-associated genes of *P. aeruginosa*.

## Methods

### **Bacterial strain**,** culture conditions and minimum inhibitory concentration (MIC)**

MICs of quercetin, baicalein, and azithromycin against *P. aeruginosa* PAO1 (ATCC^®^ BAA-47™) were determined by the microdilution method according to the Clinical & Laboratory Standards Institute, using 96-well plates. First, three 10 mg/mL stock solutions were prepared, one of ethanol for azithromycin (AK Scientific Inc^®^, Union City, CA, USA) and the other two of dimethyl sulfoxide (DMSO) for quercetin and baicalein (Sigma-Aldrich^®^, St. Louis, MO, USA). From these solutions, the dilutions to be evaluated were prepared, based on the reports in the literature [20–22], as follows: for quercetin, 250–4 µg/mL; for azithromycin, 250 –0.25 µg/mL; and for baicalein, 125 –0.25 µg/mL. The assay was performed following the methods previously reported [23]. DMSO was used as a vehicle at a final concentration of 1% (v/v), which was previously validated to have no significant effect on *P. aeruginosa* viability or growth kinetics [23].

### Selected concentrations for quercetin, baicalein, azithromycin, and their combinations

Biofilm formation was evaluated at concentrations that did not affect bacterial growth according to the susceptibility results. For virulence factor production (elastase, pyocyanin, and rhamnolipids), each compound and its combinations (quercetin–azithromycin, quercetin–baicalein, and azithromycin–baicalein) were tested at sub-MIC levels (1/16 MIC). The combination showing the greatest inhibition of biofilm formation and virulence factors, together with its individual components, was subsequently used for gene expression analyses.

### Biofilm formation

This test was conducted as in the susceptibility test. After the incubation time the non-adherent bacteria were removed by three washes with sterile PBS, while adherent biofilm-forming cells were stained with 200 µL of crystal violet (0.1% [w/v] in H_2_O) and incubated at room temperature for 15 min. Following this period five washes with PBS were done, the crystal violet was solubilized with ethanol (95%) and the absorbance was measured at 570 nm using a plate reader (Thermo Scientific™ Multiskan™). Five replicates were used for each treatment and the results were expressed as a formation percentage according to Eq. 1 [24],

1 $$\begin{aligned} \\& \:Biofilm\:formation\:\left(\%\right)= \\& \left(\frac{(Average\:absorbance\:of\:treatments\:-Average\:absorbance\:of\:controls\:of\:the\:treatments)}{(Average\:bacterial\:absorbance+DMSO-Average\:LB\:broth\:control\:absorbance+DMSO)}\right)*\:100 \end{aligned}$$

### Equation 1

Percentage of biofilm formation.

The interaction between individual compounds and their combinations was quantified using the Summed Combinatorial Index (ΣCI) [25], according to Eq. 2,

$$\:{\Sigma\:}CI=CIA\:+CIB\:$$

2 $$\:CIA=\frac{IBCA}{IBCAmixture}$$

### Equation 2

Summed Combinatorial Index.

For this calculation, the individual index of each component of the mixture was first determined, CIA and CIB. This index was obtained by dividing the result achieved by that agent individually in inhibiting biofilm formation in *P. aeruginosa* (IBCA) by the result obtained by the same compound when acting in the mixture (IBCAmixture). Finally, the type of interaction was categorized based on the ΣCI values ​​obtained as follows:

Synergy: ΣCI < 0.5, Additive effect: 0.5 ≤ ΣCI ≤ 2, Neutral effect: 2 < ΣCI ≤ 4, Antagonism: ΣCI > 4.

### Virulence factors

The supernatant was separated from the bacterial inoculum treated with quercetin, azithromycin, baicalein and their combinations by centrifugation at 8,000 × *g* for 10 min. This supernatant was used to continue with the elastase and pyocyanin production assays; while for the rhamnolipid assay it was centrifuged twice at 2000 × *g* for 10 min each cycle. Each assay was performed in triplicate and included a positive control (bacteria in LB+DMSO medium) and a blank (no treatment + LB medium).

### Elastase production

25 µL of culture supernatant were taken and mixed with 225 µL of buffer (100 mM Tris at pH 7.5) containing elastin-Congo red (ECR) (Sigma-Aldrich^®^) (10 mg/mL) and then incubated at 37 °C for 3 h under constant shaking. After incubation, it was left to cool for 2 min, 250 µL of PBS (0.7 M pH 6.0) were added to it and centrifuged at 8,000 × *g* for 10 min to remove insoluble ECR. Finally, elastase activity was measured at 495 nm [26] in Multiskan™ equipment (Thermo Scientific™).

### Pyocyanin production

750 µL of supernatant were mixed with 375 µL of chloroform using a vortex. Then, the sample was centrifuged at 8,000 × *g* for 10 min at 4 °C, the upper phase was discarded, and the organic (lower) phase was mixed with 300 µL of 0.2 M HCl until a pink coloration was observed. The sample was centrifuged once again at 8,000 × *g* for 10 min, and 150 µL of the aqueous phase were taken and diluted with 150 µL of buffer (200 mM Tris at pH 8.0). Finally, pyocyanin production was measured at 390 nm [26].

### Rhamnolipid production

1,000 µL of the culture supernatant was taken and extracted three times with 1 mL of diethyl ether. The organic phase of each extraction was pooled in one tube for each treatment and, later, this solution was dried at 35 °C and dissolved in 100 µL of water. It was then mixed with 100 µL of 1.7% orcinol and 800 µL of 60% H_2_SO_4_. For the blanks, a mixture of 100 µL of water with 100 µL of 1.7% orcinol and 800 µL of 60% H_2_SO_4_ was used. Finally, the mixture was heated to boiling for 30 min and then the absorbance was measured at 420 nm [27] in MultiskanTM equipment (Thermo Scientific™).

All absorbances were measured using a plate reader (Thermo Scientific™ Multiskan™). Results were expressed as production percentages according to Eq. 3,

3 $$\begin{aligned} \\& \:Production\:of\:the\:virulence\:factor\:\left(\%\right)= \\& \left(\frac{(Average\:absorbance\:of\:treatments\:-Average\:absorbance\:of\:controls\:of\:the\:treatments)}{(Average\:bacterial\:absorbance+DMSO-Average\:LB\:broth\:control\:absorbance+DMSO)}\right)*\:100 \end{aligned}$$

### Equation 3

Percentage of virulence factor production.

### **Gene expression assay**

An overnight culture was diluted 1:100 and subsequently exposed to the treatments, including positive and negative controls. Bacterial growth was monitored until it reached the mid-exponential phase (OD_600_ = 0.6–0.8). To stabilize the RNA, 750 µL of RNAprotect Bacteria reagent (Qiagen^®^, Hilden, Germany) was added to 375 µL of the culture, followed by incubation and centrifugation according to the manufacturer’s instructions. RNA extractions and cDNA synthesis were performed following a previously described protocol using the RNeasy mini kit (Qiagen^®^), RQ1 RNase-Free DNase (Promega^®^, Madison, WI, USA) and M-MLV Reverse Transcriptase enzyme (Promega^®^) [23]. For RT-qPCR, 2 µL of cDNA, 0.25 µM of each primer (Table 1), the 1X Luna^®^ Universal qPCR Kit, and the CFX96 real-time PCR kit (Bio-Rad) were used. The cycling parameters were one cycle at 95 °C for 5 min, 30 amplification cycles of 95 °C for 45 s (denaturation), and 62 °C for 30 s, and a final cycle corresponding to the 60–95 °C melting curve. Amplification profiles, melting curves, and cycle threshold (Ct) values for *lasR* and *mvfR* genes were analyzed and normalized against the housekeeping gene *rpoD* [28]. Relative gene expression was calculated using the 2^−∆∆Ct^ method [29].

**Table 1:** List of target genes and their corresponding forward and reverse primers

Gen	Forward Primer (5’ – 3’)	Reverse Primer (5’ – 3’)	Amplification size	Reference
*mvfR*	ACCTGGAAATCGACCTGTG	TGAAATCGTCGAGCAGTACG	238 pb	[30]
*lasR*	ACGCTCAAGTGGAAAATTGG	GTAGATGGACGGTTCCCAGA	241 pb	[30]
*rpoD*	GGGCGAAGAAGGAAATGGT	CAGGTGGCGTAGGTGGAGA	178 pb	[31]

### Statistical analysis

Susceptibility and biofilm formation assays were performed in quintuplicate, while virulence factors (elastase, pyocyanin, and rhamnolipid) and gene expression (*lasR* and *mvfR)* assays were conducted in triplicate. The mean, standard deviation, and coefficient of variation were determined for each assay. Statistical analyses were performed using RStudio and all figures were generated in Microsoft Excel. Data were evaluated by one-way analysis of variance (ANOVA) followed by Tukey’s post-hoc test for multiple comparisons. Differences relative to the *P. aeruginosa* PAO1 positive control were considered statistically significant at *p* ≤ 0.05 and *p* ≤ 0.01.

## Results

### Determination of MIC of quercetin, baicalein, and azithromycin in *P. aeruginosa*

The susceptibility profile of *P. aeruginosa* PAO1 revealed MIC values of 16 µg/mL for azithromycin and 62 µg/mL for baicalein, defined as the lowest concentrations that completely suppressed visible bacterial growth after 24 h. Regarding quercetin, the MIC was determined to be 250 µg/mL. Although a slight reduction in turbidity was noted at the highest tested concentration (250 µg/mL), higher doses could not be evaluated due to compound precipitation, which interferes with accurate spectrophotometric readings. At their respective MICs, azithromycin and baicalein achieved growth inhibition levels of 49% and 48% (***p ≤ 0.001), demonstrating a significant impact on bacterial. Notably, both compounds maintained a substantial inhibitory effect even at sub-MIC levels (Table 2), providing a robust baseline for evaluating their anti-virulence potential without inducing high selective pressure.

**Table 2:**
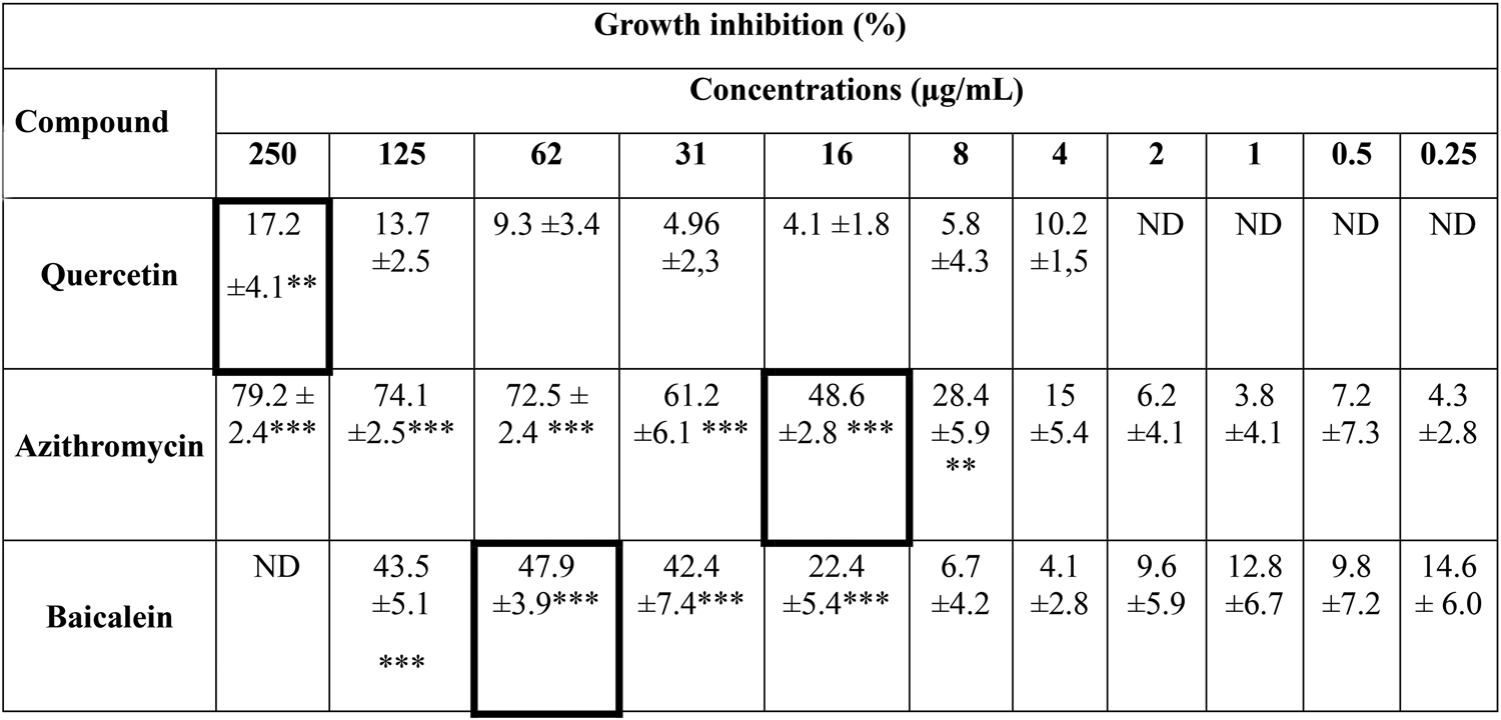
Susceptibility of *P. aeruginosa* PAO1 to the evaluated compounds at the tested concentrations, expressed as percentage of growth inhibition (mean ± standard deviation) relative to the untreated control. Statistical significance was determined by comparison with the untreated control (bacteria grown in medium containing only the solvent)

Significance levels are indicated as * *p* ≤ 0.5 y ** *p* ≤ 0.01 y ***p ≤ 0.001. Cells highlighted with black borders indicate the lowest concentration at which no visible turbidity associated with bacterial growth was observed (visual MIC determination). ND indicates not determined

The determination of the minimum inhibitory concentration (MIC) allowed the establishment sub‑inhibitory concentrations (Sub‑MIC), such as 1/2, 1/4 or lower doses, to ensure that the effects observed in subsequent experiments resulted from the interference with bacterial communication systems rather than from direct antimicrobial activity or the elimination of the pathogens. Although growth kinetics were not assessed, experimental validation was supported by monitoring optical density (OD) at the end of the assays (typically at 24 h), confirming that the final biomass of the treated groups did not present significant differences compared with the control [18]. In addition, it was verified that the evaluated molecules complied with the acceptable threshold established for this class of inhibitors, whereby interference with bacterial growth should not exceed 20%, thus avoiding selective pressure that could promote the development of antimicrobial resistance as determined in previously reported in the literature [12].

### Evaluation of *P. aeruginosa* biofilm formation in response to quercetin, baicalein and azithromycin

**Fig. 1:**
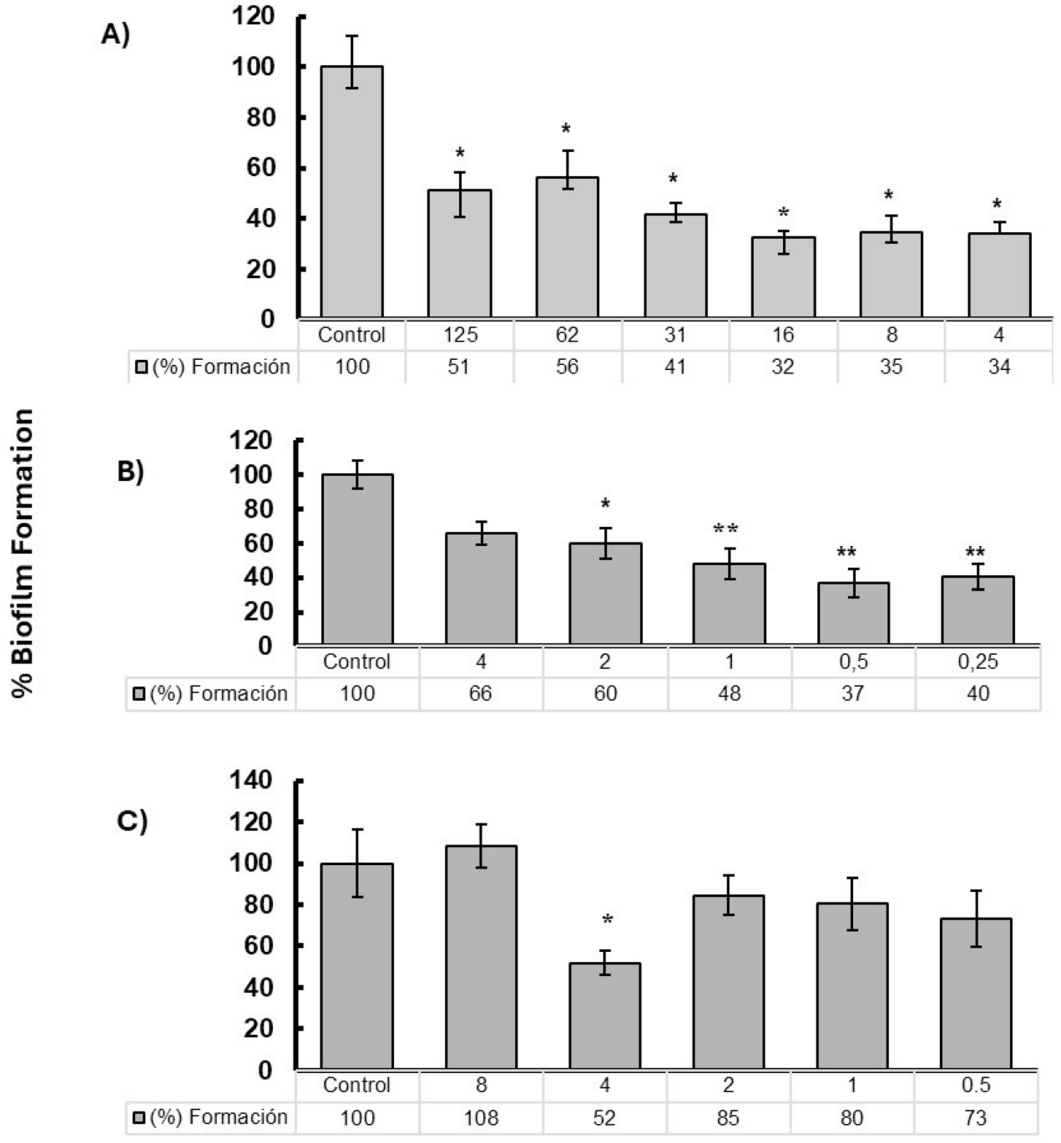
Effect of treatments on *P. aeruginosa* PAO1 biofilm formation. **a**) quercetin; **b**) azithromycin; **c**) baicalein. Values in the first row of the chart indicate the final concentration of each compound used (μg/mL). Error bars show standard deviations. *p ≤ 0.05 and **p ≤ 0.01 compared to the *P. aeruginosa* PAO1 control

A significant reduction in biofilm formation was observed in response to quercetin treatments starting at the 125 µg/mL. Concentrations of 16, 8, and 4 µg/mL showed the greatest reduction compared to the control group, with formations of 32%, 35%, and 34%, respectively (*p* ≤ 0.05) (Fig. 1a). Results for azithromycin showed a significant reduction in biofilm formation from concentrations of 2, 1, 0.5, and 0.25 µg/mL, with formation percentages of 60%, 48%, 37%, and 40%, respectively (*p* ≤ 0.01) (Fig. 1b). In the case of baicalein at 4 µg/mL, biofilm formation was significantly reduced to 52% compared to the control group (*p* ≤ 0.05) (Fig. 1c).

### Evaluation of the effect of quercetin-baicalein, quercetin-azithromycin and azithromycin-baicalein combinations on *P. aeruginosa* biofilm formation

**Fig. 2:**
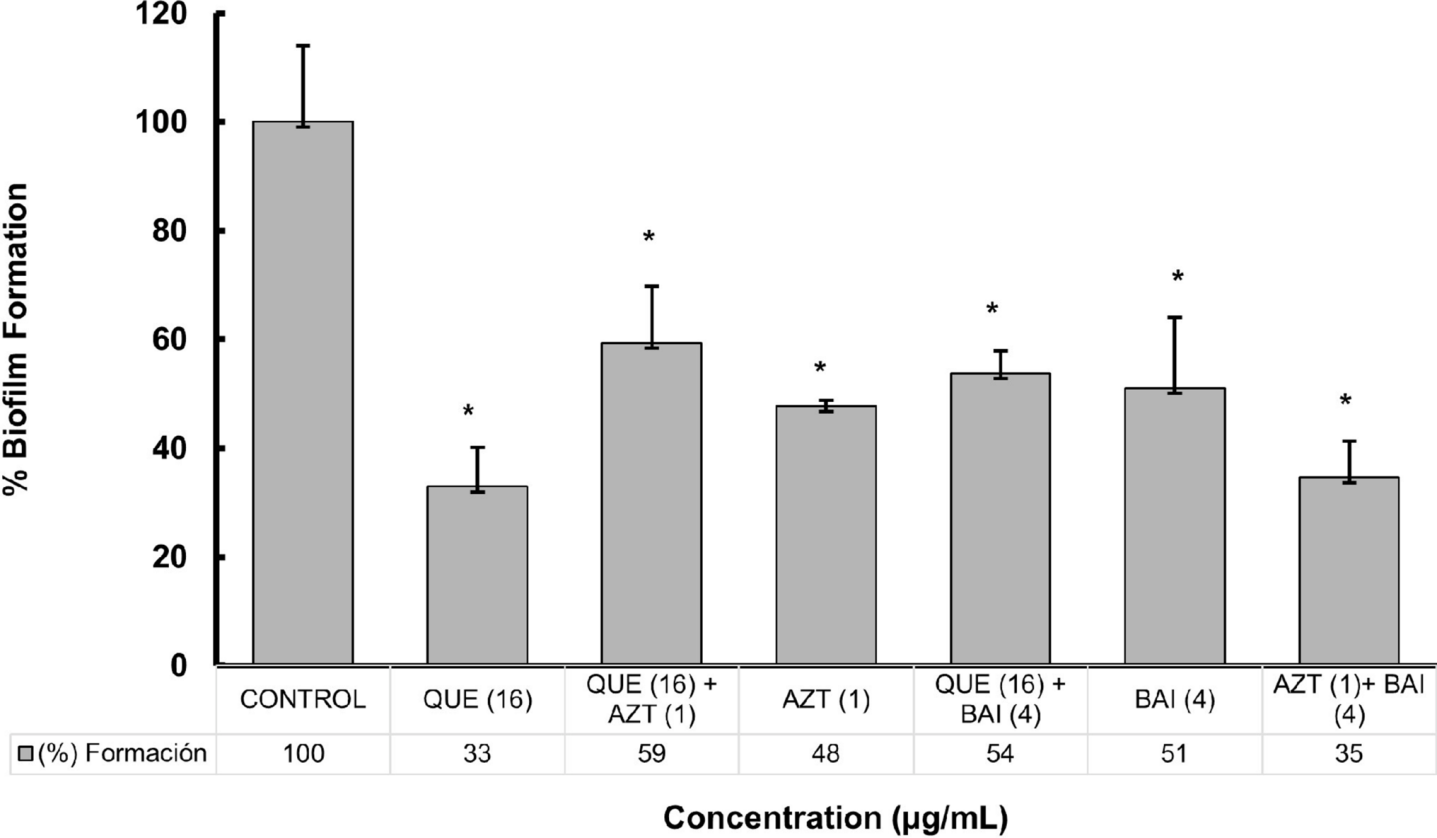
Effect of quercetin (QUE), azithromycin (AZT) and baicalein (BAI) and combinations on *P. aeruginosa* PAO1 biofilm formation. Values in parentheses indicate the final concentration of each compound used (µg/mL). Error bars show standard deviations. *p ≤ 0.05 compared to the *P. aeruginosa* PAO1 control. Concentrations used for each treatment are in parentheses

Regarding biofilm formation, individual quercetin and the combination of azithromycin with baicalein showed the best effect, with the lowest formation values of 33% and 35%, respectively, compared to the control. The azithromycin-baicalein mixture exhibited better effects than the individual compounds **(**Fig. 2**).** In contrast, the combinations of quercetin with baicalein, and quercetin with azithromycin showed a decrease in their effect compared to the individual compounds. It is important to mention that the three combinations had no significant difference in the growth of *P. aeruginosa* PAO1 (NS, *p* > 0.05), (Data not shown).

Regarding the Combinatorial Index (∑CI), the analysis revealed that mixtures including quercetin (the most potent individual inhibitor, with a 67% inhibition) exhibited a reduction in biological efficacy, achieving inhibition levels of only 41% and 46% in mixtures quercetin- azithromycin and quercetin-baicalein, respectively. These combinations yielded ΣCI values of 2.90 and 2.50, and were therefore categorized as indifferent interactions (range 2.0–4.0). This suggests that the presence of other agents negatively interferes with the activity of quercetin.

In contrast, the combination of azithromycin-baicalein, demonstrated an improved performance compared to the individual compounds (52% and 48%, respectively), reaching a 65% inhibition when combined. The ΣCI calculated for this mixture was 1.54, which corresponds to an additive effect (range 0.5–2.0), confirming a functional cooperation between both compounds, although not reaching the threshold required to be classified as a synergistic interaction (ΣCI < 0.5).

### Effect on the production of virulence factors of *P. aeruginosa* PAO1 in response to quercetin, baicalein, azithromycin and their combinations

**Fig. 3:**
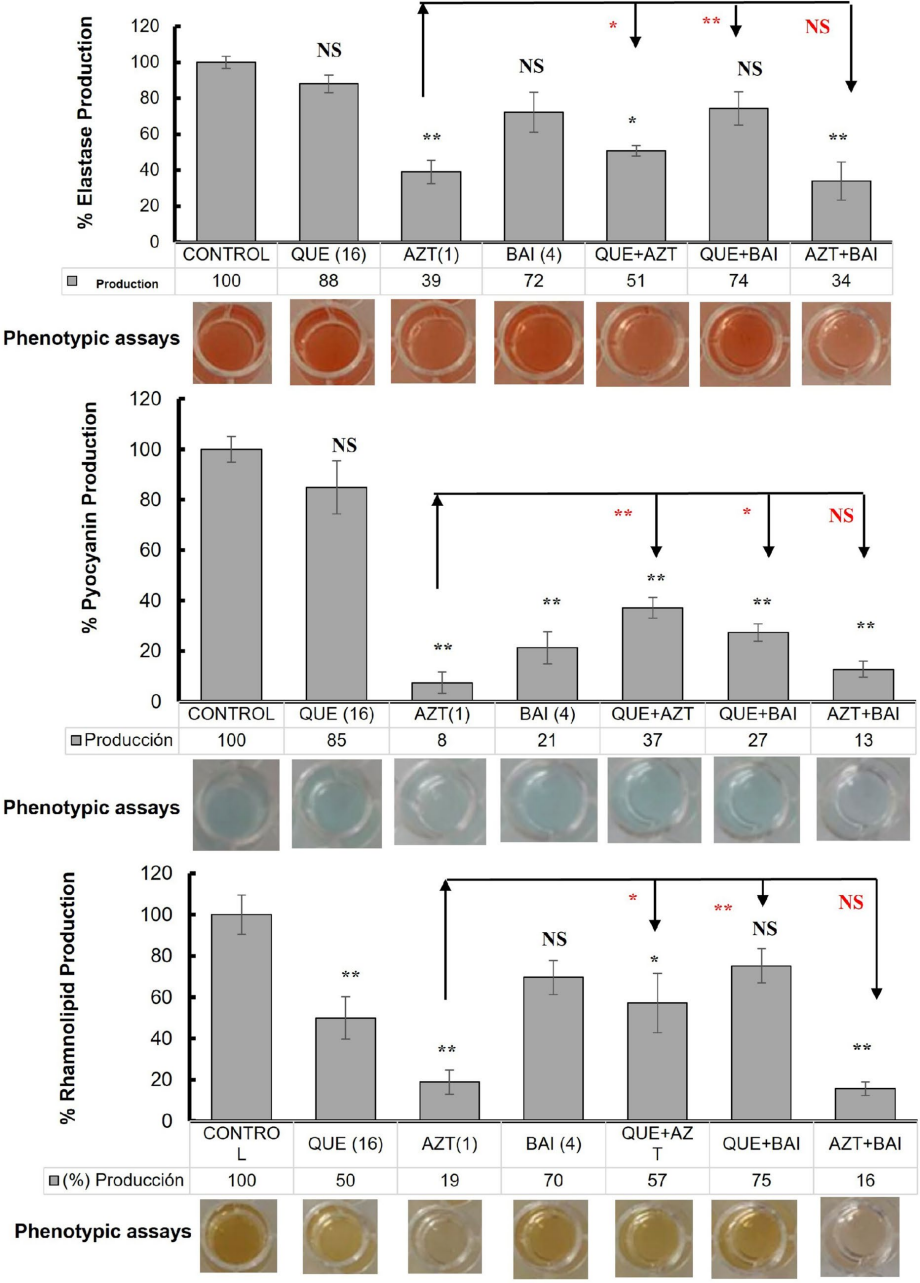
Sub-MIC inhibitory effect of quercetin, azithromycin, baicalein and their combinations on the production of **a**) Elastases; **b**) Pyocyanin and **c**) Rhamnolipids in *P. aeruginosa* PAO1. The bar charts represent the quantitative production percentages relative to the control. Values in parentheses indicate the final concentration of each compound used (µg/mL). Representative photographs of the corresponding phenotypic assays are displayed below each chart to illustrate qualitative changes in pigment and metabolite production. Statistical significance: NS, *p* > 0.05; * *p* ≤ 0.05; ** *p* ≤ 0.01, where black asterisks show the comparation with control *P. aeruginosa* PAO1 and red asterisks the comparation with the best performing single compound

The compound with the greatest reduction in the production of elastases, pyocyanin and rhamnolipids was azithromycin with 39%, 8%, and 19% production, respectively (*p* ≤ 0.01). In the case of quercetin and baicalein, they showed a significant difference compared to the PAO1 control in rhamnolipids and pyocyanin production (*p* ≤ 0.01). As for the combinations, azithromycin with baicalein had the highest reduction of elastases, pyocyanin and rhamnolipids with 34%, 13%, and 16% compared to the other mixtures, being significantly different between them (*p* ≤ 0.05) (Fig. 3). Likewise, this combination proved to be statistically equal to the individual compound with the best results, corresponding to azithromycin (NS, *p* > 0.05), for the three evaluated factors, and is different from the individual compound baicalein for elastases and rhamnolipids production, in which it did not reduce the production of these two factors significantly (*p* ≤ 0.01).

On the other hand, the combination of quercetin with azithromycin had a significant reduction compared to the control of elastase, pyocyanin, and rhamnolipid production with 51%, 37% and 57% production, respectively (*p* ≤ 0.01) (Fig. 3). However, its effect was lower than azithromycin and quercetin alone for all three virulence factors (*p* ≤ 0.05) (Fig. 3**)**. Additionally, it was observed that the combination of quercetin with baicalein had no effect on elastase and rhamnolipid production (NS, *p* > 0.05), but present on pyocyanin with 27% production (Fig. 3b), which, compared to its individual compounds, only had difference compared to quercetin (*p* ≤ 0.01).

### Effect of baicalein, azithromycin and their combination on the expression of *mvfR* and *lasR* genes of *P. aeruginosa*

**Fig. 4:**
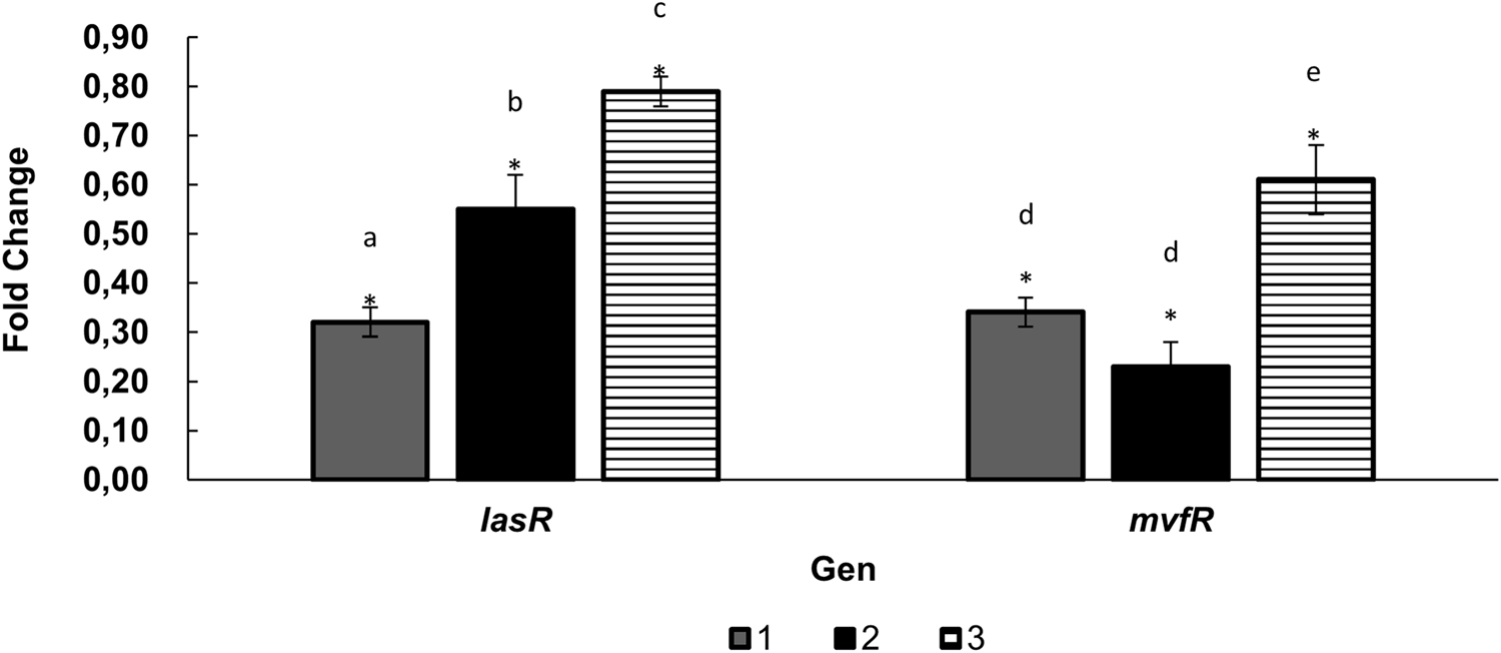
Gene expression of *lasR* and *mvfR* genes of *P. aeruginosa* PAO1 by RT-qPCR in response to the combination of azithromycin with baicalein (1), azithromycin (2), and baicalein (3) in comparison to the control *P. aeruginosa* PAO1. The *rpoD* gene was used as a calibrator and the expression was normalized by the 2-ΔΔΔCT method. *Treatments significantly different from control. Lower-case letters (a–e) represent statistical groupings within each gene; means sharing the same letter are not significantly different (one-way ANOVA followed by Tukey’s HSD test; *p* < 0.05)

Among the evaluated mixtures, the baicalein-azithromycin combination demonstrated the highest efficacy in reducing both biofilm formation and the production of virulence factors. Consequently, the individual compounds and the mixture were assessed for their effects on the expression of the *mvfR* and *lasR* genes in *P. aeruginosa*. In RT-qPCR all treatments had a significant effect, however, the combination of azithromycin with baicalein showed the best decrease in *lasR* gene expression with a percentage of 32% (*p* ≤ 0.05), followed by azithromycin with an expression percentage of 55% compared to the control (*p* ≤ 0.05) (Fig. 4). In *mvfR* expression, azithromycin, the combination azithromycin-baicalein and baicalein had 24%, 34% and 61% expression, (*p* ≤ 0.05) respectively, the first two treatments being significantly equal.

## Discussion

This study evaluated the effects of quercetin and baicalein (flavonoids), the antibiotic azithromycin, and their combinations on biofilm formation, virulence factor production, and QS gene expression in *P. aeruginosa*. The MIC of quercetin and azithromycin were determined to be > 250 and 16 µg/mL, respectively, which are consistent with previously reported values [22, 32]. In contrast, the MIC obtained for baicalein (62 µg/mL) differs from earlier reports indicating that concentrations > 256 µg/mL were required to produce significant inhibition of bacterial growth and cell density [21, 33]. This discrepancy may be attributed to differences in experimental conditions, such as the culture medium employed, among other methodological variables.

At the concentrations evaluated, baicalein (4 µg/mL) and quercetin (16 µg/mL) did not exhibit toxicity or bactericidal effects and are considered non-toxic based on previous literature reports [12, 34, 35]. Baicalein has not shown adverse effects in vivo toxicity models, such as *Caenorhabditis elegans*, even at doses up to 250 ppm. Furthermore, pharmacokinetic and toxicological analyses, including the Ames test, have confirmed the absence of mutagenic or carcinogenic properties. Quercetin is notable for its ubiquitous presence in human diet products (fruits and vegetables) and has been reported to possess cytoprotective activities, protecting host cells rather than damaging them or inducing malignant transformations [34]. These flavonoids have been classified as safe for human consumption (GRAS status). Consequently, at the tested concentrations, these compounds function exclusively as anti-virulence agents, precluding selective pressure on the bacteria and ensuring no direct cellular toxicity.

The results of this research study showed a lower percentage of biofilm formation for quercetin, followed by the azithromycin-baicalein combination, of 33% and 35%. Quercetin inhibition of biofilm formation may be related to LasR inhibition, which controlled *rhlR* expression and plays an important role in these processes [4]. The relevance of LasR and MvfR in biofilm formation has been well documented, as *P. aeruginosa* strains deficient in *lasI* or *mvfR* produce less structured biofilms with reduced biomass and viability. These findings support LasR and MvfR as promising anti-biofilm targets, because inhibition was observed with the azithromycin–baicalein combination, and with quercetin, azithromycin, and baicalein individually.

Flavonoids have been widely reported as QS inhibitors, through multiple mechanisms, including competitive inhibition by occupying the ligand binding site, by inhibiting protein stability, solubility or dimerization, and by affecting DNA binding or RNA polymerase engagement [36]. In particular, quercetin-mediated biofilm inhibition has been linked to its hydroxylated structure, which alters membrane permeability and promotes leakage of cellular components. Additionally, quercetin can disrupt the bacterial membrane, degrade the extracellular biofilm matrix, inhibit efflux pumps, interfere with cell envelope formation, modulate quorum sensing pathways, and chelate iron, thereby reducing bacterial adhesion and proliferation [37]. In this context, the potential of flavonoids may be exploited to enhance the efficacy of antibiotics when used in combination. For this reason, in this study, two new combinations were proved, quercetin with baicalein and quercetin with azithromycin. This antibiotic interferes with the Gac/Rsm system, which positively controls quorum sensing in *P. aeruginosa*. Based on the results of this study, it is determined that the combinations of quercetin with azithromycin and quercetin with baicalein did not exhibit greater effects than the individual compounds, but this study represents the first report evaluating these specific combinations.

The Summed Combination Index showed indifferent and additive effects with the mixtures studied. These results are consistent with patterns previously reported in studies evaluating the use of compound mixtures for biofilm control. For instance, additive effects have been reported for combinations of benzaldehydes with antibiotics or naringenin with ciprofloxacin [25, 38], whereas indifferent effects have been described for various combinations of polyphenols with amino acids [12].

The potential of baicalein combinations with other antibiotic has been reported previously, for example baicalein in combination with ceftazidime on infections of *P. aeruginosa* in the intraperitoneal cavities of mice, showed greater reduction in bacterial colony counts, thinner biofilms and less bacterial adhesion, compared to the groups that received the treatments individually or the control . The potential benefit of the combination may be attributed to the activity of baicalein alone, which reduces the number of *P. aeruginosa* PAO1 cells adhering to abiotic surfaces during the early stages of colonization, thereby highlighting its capacity to interfere with the initial steps of biofilm formation. These findings are supported by scanning electron microscopy (SEM) analyses, which revealed structural alterations in the biofilm architecture, resulting in less dense and mechanically weaker biofilms [33].

The potential of baicalein has led to the development of gold nanoparticles functionalized with this flavonoid that has shown notable antibiofilm activity against *P. aeruginosa* PAO1 and decreased the production of exopolysaccharides (EPS). Microscopy studies revealed that treated biofilms exhibited a less dense and thinner structure. Therefore, the potential of this compound has been demonstrated to be a promising strategy against chronic biofilm-associated infections [39].

Among the virulence factors evaluated, azithromycin produced the most pronounced reduction across all parameters, whereas quercetin selectively decreased rhamnolipid production and baicalein mainly affected pyocyanin levels. All combinations reduced virulence factor production, with azithromycin–baicalein showing the strongest overall effect. These findings are consistent with previous reports demonstrating that azithromycin, at subinhibitory concentrations, suppresses motility and the production of multiple virulence determinants in *P. aeruginosa*, including proteases, pyocyanin, exotoxin A, phospholipase C, and exopolysaccharides [40]. This activity has been linked to quorum sensing inhibition, as azithromycin reduces the synthesis of the signal molecules 3OC12-HSL and C4-HSL through transcriptional repression of the *lasI/lasR* and *rhlI/rhlR* systems [41].

In the present study, baicalein inhibited pyocyanin production, similarly to a previous report where higher subinhibitory concentrations (> 125 µg/mL) attenuated the production of extracellular virulence factors in *P. aeruginosa* PAO1, such as pyocyanin (69.87% reduction) [33]. In this report baicalein, generated greater inhibition of LasA protease, LasB elastase, and rhamnolipid than in the present study, this could be attributed to the different concentrations evaluated. The antivirulence activity of baicalein has been associated with reduced quorum sensing signal production and can be reversed by supplementation with exogenous AHLs, supporting its role as a QS inhibitor without directly killing the bacteria [33, 42]. Notably, this study represents the first report describing the effect of azithromycin combined with baicalein on virulence factor production. Comparable synergistic antivirulence effects have been reported for combinations of antibiotics with antivirulence agents or natural compounds, including colistin or tobramycin combined with gallium or furanone C-30, as well as ceftazidime–thymol and norfloxacin–curcumin, highlighting the potential of combination strategies to attenuate *P. aeruginosa* pathogenicity [43].

Several studies have demonstrated that natural compounds can modulate quorum sensing (QS) systems in *P. aeruginosa* by affecting the expression of key regulatory genes such as *lasR* and *mvfR*. In this context, the present study provides the first evidence that the combination of baicalein and azithromycin significantly modulates these QS systems, resulting the greatest downregulation of *lasR* expression (32%; *p* ≤ 0.05). Azithromycin alone also significantly decreased *lasR* expression (55%; *p* ≤ 0.05), confirming its QS-modulating activity, in agreement with previous reports describing up to a sevenfold reduction in *lasR* expression. This effect is consistent with earlier observations showing that azithromycin reduces *lasR*-dependent reporter gene expression, leading to the downregulation of not only the Las system but also the subordinate Rhl and MvfR pathways [44]. Previous reports have shown the efficacy of baicalein alone and in combination with antibiotics in modulating QS in *P. aeruginosa* 33]. The results of this research also supported the individual efficacy of the evaluated compounds, even at lower concentrations than previously reported. These findings are promising, as they could potentially reduce the emergence of bacterial resistance, side effects, or toxicity associated with high drug doses [45].

Biofilm formation and virulence factor production in *P. aeruginosa* are tightly regulated by quorum sensing (QS) genes such as *lasR* and *mvfR*, which are considered promising therapeutic targets [4]. In this study, the azithromycin–baicalein combination exerted a global inhibitory effect on QS signaling, as evidenced by the downregulation of *lasR* and *mvfR* and the concomitant reduction in QS-regulated virulence factors, including biofilm formation, elastase activity, pyocyanin, and rhamnolipid production. These findings are consistent with the central role of the LasI/LasR system in controlling elastase production and regulating the downstream *rhlR* pathway, which governs rhamnolipid synthesis [42]. In parallel, inhibition of *mvfR* likely contributed to the observed decrease in pyocyanin and rhamnolipids, in line with its broader involvement in biofilm development, iron acquisition, toxin production, and antibiotic resistance in *P. aeruginosa* [46]. While the present study provided insights into the modulation of key regulatory genes, it is important to acknowledge that the QS network in *P. aeruginosa* functions as a complex, interconnected hierarchy. A more comprehensive understanding of the inhibitory mechanism of these flavonoids would require the evaluation of signaling genes such as *lasI*, *rhlI/R* and *pqs*ABCDE. Assessing these genes in future studies will be essential to fully elucidate how these compounds interfere with the entire signaling cascade and to determine their impact on the global transcriptional profile of the pathogen.

The observed attenuation of biofilm formation and the significant reduction in the production of virulence factors (such as pyocyanin, elastase, and proteases) in this study are closely correlated with the transcriptional repression of the regulatory genes as lasR and mvfR. Previous studies have demonstrated that this phenotypic and genotypic inhibition is mechanistically supported by molecular docking analysis. These studies have reported that flavonoids frequently act as competitive or non‑competitive antagonists at the binding sites of quorum‑sensing (QS) receptors [10]. Among these compounds, baicalein (in its aglycone form) generally exhibits some of the most competitive docking scores reported, although its relative affinity varies depending on the specific QS receptor evaluated. The high affinity of baicalein for the LasR, RhlR and MvfR receptors consistently positions it as a broad‑spectrum, high‑affinity inhibitor, outperforming even its glycosylated form, baicalin [47]. From a structural perspective, the potency of baicalein is attributed to the presence of three adjacent hydroxyl groups on ring A, which promote the formation of complex hydrogen‑bonding networks and π–π stacking interactions with key residues. In particular, interactions with TRP60, ARG61 and THR75 have been described in LasR, while in PqsR baicalein forms a hydrogen bond with SER196 and establishes hydrophobic contacts with residues such as PHE221 and TYR258 [12, 47].

In the case of quercetin, available studies indicate that it primarily acts through a non‑competitive allosteric mechanism. Quercetin has been computationally validated as a potent LasR antagonist, capable of occupying the binding site of the natural autoinducer (3‑oxo‑C12‑HSL) and inducing conformational rearrangements in the receptor (8). However, despite azithromycin being widely recognised for its QS‑antagonistic activity and its ability to reduce the production of signalling molecules, no specific docking studies have been reported that directly compare it or evaluate it in combination with baicalein or quercetin, highlighting the need for such analyses. This gap in the literature can be partly explained by the fact that azithromycin exerts its antivirulence effects primarily through transcriptional repression of the regulatory genes lasI/R and rhlI/R, as well as by interfering with protein translation at the ribosomal level, rather than acting as a direct competitive antagonist at autoinducer binding sites [44].

In conclusion, the findings presented in this study constitute a fundamental preliminary approach to identification of quorum sensing inhibitors, employing the PAO1 strain as a biological reference model. This strain, originally isolated from an infected wound, is widely used in laboratory settings due to its well-characterized resistance profile, which combines intrinsic resistance mechanisms with the capacity for horizontal gene acquisition. These features have established PAO1 as a robust in vitro model for investigating pathogenicity and antimicrobial resistance in *P. aeruginosa*, and its extensively sequenced genome and phenotypic stability enable reproducible validation [48]. Nevertheless, as a future perspective and essential recommendation, further evaluation using in vivo systems, such as the *Caenorhabditis elegans* model, and clinical isolates is required to fully assess the translational and therapeutic potential of these QS inhibitors.

## Conclusion

Based on the findings presented in this study, it was observed that quercetin, azithromycin and the azithromycin-baicalein combination show significant potential to control biofilm formation and virulence factors associated with *P. aeruginosa*. Specifically, these compounds and their combinations showed notable reductions in the percentages of biofilm formation, elastase, pyocyanin and rhamnolipid production, as well as inhibition of quorum sensing gene expression.

Among the treatments, azithromycin and the azithromycin-baicalein combination stood out for their efficacy in inhibiting biofilm formation and virulence factors, showing a remarkable inhibition in gene expression related to quorum sensing regulation. Importantly, the combination of azithromycin and baicalein showed the most promising results, suggesting possible routes for future antimicrobial therapies.

## Data Availability

No datasets were generated or analysed during the current study.
